# Blood Supplementation Enhances Bartonella henselae Growth and Molecular Detection of Bacterial DNA in Liquid Culture

**DOI:** 10.1128/spectrum.05126-22

**Published:** 2023-05-25

**Authors:** Chance Liedig, Pradeep Neupane, Erin Lashnits, Edward B. Breitschwerdt, Ricardo G. Maggi

**Affiliations:** a Intracellular Pathogens Research Laboratory, Department of Clinical Sciences, and the Comparative Medicine Institute, College of Veterinary Medicine, North Carolina State University, Raleigh, North Carolina, USA; b Department of Microbiology and Immunology, School of Medicine, University of North Carolina, Chapel Hill, North Carolina, USA; c Department of Medical Sciences, School of Veterinary Medicine, University of Wisconsin-Madison, Madison, Wisconsin, USA; Johns Hopkins Medicine

**Keywords:** BAPGM, *Bartonella* culture, *Bartonella henselae*, bartonellosis, blood supplementation, diagnostics

## Abstract

Bacteria of the genus *Bartonella*, a member of the *Alphaproteobacteria*, are fastidious, Gram-negative, aerobic bacilli that comprise numerous species, subspecies, and genotypes. Bartonella henselae, with a worldwide distribution, infects cats, dogs, horses, humans, and other mammals. Diagnostically, direct detection of Bartonella henselae in patient blood specimens by culture or molecular methods is required to confirm infection with this bacterium. Enrichment blood culture combined with quantitative PCR (qPCR) or ddPCR enhances the sensitivity of direct detection. The addition of sheep blood to liquid culture media increased the Bartonella henselae DNA concentration compared to controls, additionally improving PCR direct detection sensitivity.

**IMPORTANCE** This study aims to improve diagnostic detection of Bartonella henselae. Patient samples are combined with enriched bacterial cultures aimed at growing Bartonella henselae for the best possible chance at detection. However, current *Bartonella* growth methods could be improved. The DNA extraction method used by most laboratories should also be optimized. Sheep blood was added to increase the growth of Bartonella henselae and multiple DNA extraction methods were to be compared to each other.

## INTRODUCTION

*Bartonella* species comprise emerging, zoonotic, bacterial pathogens that are of increasing importance in human and veterinary medicine. Bartonella henselae, most often transmitted by *Ctenocephalides felis* (a flea species that is widely distributed throughout much of the world) is likely the most important cause of bartonellosis in cats, dogs, horses, and humans (1–11). Therefore, studies related to B. henselae, the main etiological agent of cat scratch disease (CSD, an acute onset illness characterized by fever, lymphadenopathy, and history of a cat scratch), are of increasing comparative biomedical importance (12–16). In addition to CSD, B. henselae bacteremia has also been associated with chronic cardiovascular, neurological, and rheumatologic symptoms in immunocompetent patients, including endocarditis, osteomyelitis, neuroretinitis, and neuropsychiatric symptoms (17–28). These bacteria seemingly induce diverse forms of pathology in immunocompetent persons, as well as bacillary angiomatosis, bacillary peliosis, and endocarditis in immunocompromised individuals (17–21, 25, 29–37). Transmission of B. henselae from cats to humans occurs most often through contamination of cat scratches with flea excrement (38). Transmission may also occur through flea bites or cat bites, if cat blood or flea excrement contaminates the bite site or viable organisms are in the cat’s mouth (11, 39).

Because of the increasing importance of B. henselae as an emergent vector borne pathogen, efforts to confirm infection are of substantial diagnostic importance in both human and veterinary medicine. Despite the acute nature of CSD, variable IgM and IgG sensitivity and specificity have been reported for serology in diagnosis of this disease in (40–43). Interpretation of B. henselae serology is substantially more complicated and problematic when testing chronically ill patients, in whom antibody titers can be low or negative despite documentation of infection by culture or PCR amplification of B. henselae DNA from blood or other tissue specimens (17, 18, 20, 21, 24, 25, 28, 29, 44–48). Recently, the sensitivity and specificity of multiple indirect fluorescent antibody (IFA) assays, western immunoblotting (WB), quantitative PCR (qPCR), and droplet digital PCR (ddPCR) using blood, serum, and tissue samples from dogs diagnosed with hemangiosarcoma was reported (49). In addition to confirming the lack of a “gold standard test” for the diagnosis of chronic *Bartonella* spp. infection in dogs, that study further emphasized the limitations associated with serologic testing (IFA and WB). It also emphasized the limitations for amplification of *Bartonella* DNA from blood, though ddPCR proved to be more sensitive than qPCR from blood samples (36% for ddPCR compared to 0% for qPCR) (49).

Previously, we reported improved blood culture diagnostic sensitivity for the detection of *Bartonella* spp. bacteremia when using an optimized insect biochemical composition growth medium (*Bartonella* alpha proteobacteria growth medium or BAPGM) (10, 34, 47, 48, 50–55). The primary objective of culturing blood (or other biological fluids) in BAPGM was to increase (or “enrich”) the number of *Bartonella* organisms to a detectable level for successful qPCR amplification and DNA sequence confirmation of the infecting species, subspecies, or strain type (8, 21, 46, 51, 52, 55–57). The BAPGM enrichment blood culture approach involved the inoculation of 2 mL of patient blood or other biological fluid sample in 10 mL of BAPGM supplemented with 1 mL of defibrinated sheep blood. To further increase the sensitivity of *Bartonella* DNA amplification from patient blood samples, without sacrificing specificity, we recently combined BAPGM enrichment blood culture with droplet digital PCR (BAPGM enrichment ddPCR) amplification of *Bartonella* spp. DNA (58, 59).

Since our initial publication described using BAPGM as a bacterial enrichment growth method, other researchers have documented enhanced blood culture diagnostic sensitivity using BAPGM, or similar insect or mammalian cell culture-based media prior to PCR testing (52, 60–66). On a clinical research basis, the use of BAPGM enrichment culture followed by qPCR (BAPGM ePCR) has facilitated the documentation of active infection with B. henselae and other *Bartonella* spp. in healthy blood donors (63, 65–67), occupationally at-risk veterinary workers (46, 51), and research study participants with a spectrum of chronic illnesses (8, 17, 20, 25, 29, 37, 68). *Bartonella* detection from blood can also be enhanced by testing 3 serial patient blood samples, obtained during a 7 day period (69). This triple blood draw approach, in conjunction with PCR testing of blood, serum, and BAPGM enrichment cultures collected at 7, 14, and 21 days (referred to as the BAPGM enrichment blood culture/PCR platform), clearly improved our ability to document *Bartonella* bacteremia by PCR amplification and DNA sequence confirmation (65). Based on2022 research testing (unpublished data, Intracellular Pathogens Research Laboratory), over 63% of human *Bartonella* spp. infections (IRB# 1960, Detection of *Bartonella* Species in the Blood of Healthy and Sick People) would not have been detected without an enrichment culture method used in conjunction with qPCR or ddPCR positive DNA results (Table 1, data from the NCSU-IPRL).

**TABLE 1:** *Bartonella* DNA detection in chronically ill people suspected of *Bartonella* infection (152 blood samples tested through the BAPGM ePCR platform [64, 66–68]). The table shows the number of samples that were positive from blood and after blood culture (at either at 7, 14 or 21 days); samples that were positive only in blood, but not after the same blood sample was cultured; and samples that were negative when the blood was tested, but were positive after the same blood sample was cultured

Blood+ & blood cult+	Blood+ & blood cult-	Blood- and Blood Cult+
12	13	44
17.4%	18.8%	63.8%

However, diagnostically, the BAPGM ePCR platform remains cumbersome due to the need for blood collection, often on alternate days for 3 collections, and costly because the microbiological testing approach requires a long incubation period (up to 21 days) to enhance the growth of *Bartonella* above the qPCR detection limit, and numerous DNA extractions to achieve a positive PCR result. Although the triple draw BAPGM ePCR platform substantially improved *Bartonella* spp. DNA detection sensitivity, it also generated numerous PCR negative data points in people who were PCR positive in only 1 or more blood or 7, 14, or 21 day culture DNA extractions (18, 20, 22, 24, 25, 31, 37, 45–47, 50, 51, 68, 70–75). Triple draw culture data clearly supported the need for further improvements in enrichment culture platform, to facilitate documentation of *Bartonella* bacteremia (bacterial DNA amplification) in patients with endocarditis and chronic cardiovascular, neurologic, and rheumatologic illnesses.

Importantly, recent studies have shown that *Bartonella* spp. form biofilms, particularly in association with heart valve infection (endocarditis), various types of surgical implants, and by potentially localizing within other sites in the body, such as bone (osteomyelitis) or the oral cavity (76–84). Unfortunately, when bacterial biofilms are present, extraction of high-quality nucleic acids for molecular analysis presents specific challenges (85–93). If, or the extent to which, biofilm formation could adversely influence culture isolation, or *Bartonella* spp. DNA amplification, has not been thoroughly investigated.

Although some blood-free media have shown good potential for the growth of B. henselae or *B. quintana* (62, 94, 95), supplementation with blood, erythrocyte membranes, or a heme component is required to achieve the full growth promoting effect of the culture medium (33, 36, 96). In the present study, we further evaluated the influence of blood supplementation on the growth of Bartonella henselae in BAPGM as medium for *Bartonella* enrichment blood culture, as compared with Brugge (Dulbecco's Modified Eagle Medium [DMEM]), a mammalian cell culture liquid medium.

## RESULTS

### Sheep blood supplement and negative controls.

*Bartonella* spp. was not cultured, and DNA was not qPCR amplified from the sheep blood used for this experiment or from any negative (naive) BAPGM or Brugge culture flask aliquot (results not shown).

### Effect of sheep blood supplementation.

**FIG 1:**
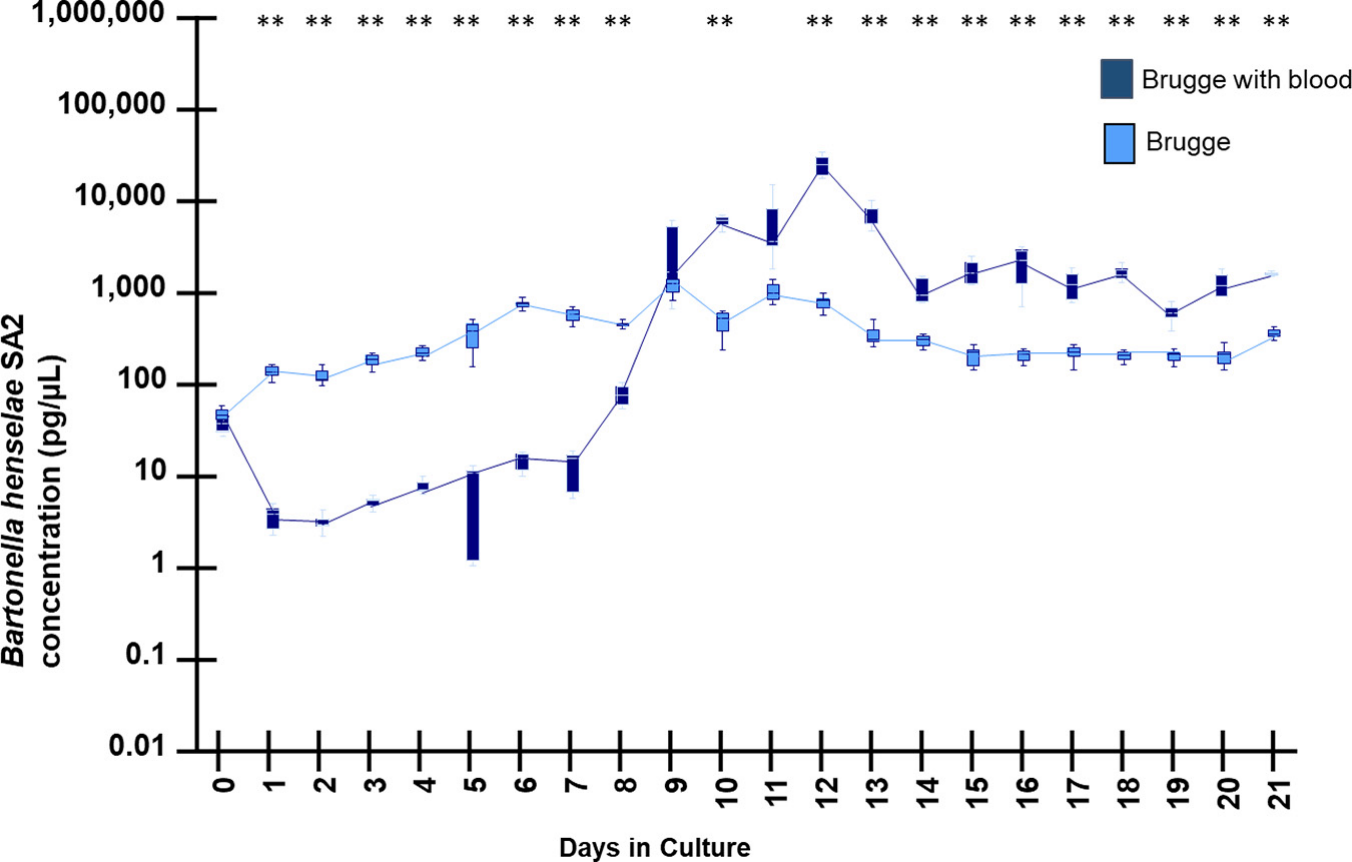
Growth kinetics determined by qPCR amplification of B. henselae SA2 DNA when cultured in Brugge medium with and without sheep blood supplementation. DNA extractions used for qPCR testing in this figure were generated using the QIAsymphony DSP DNA kit. Box and whiskers plot displaying variation in the median, the first and third quartiles, and the minimum and maximum values obtained. ** denotes *P* < 0.01 values.

**FIG 2:**
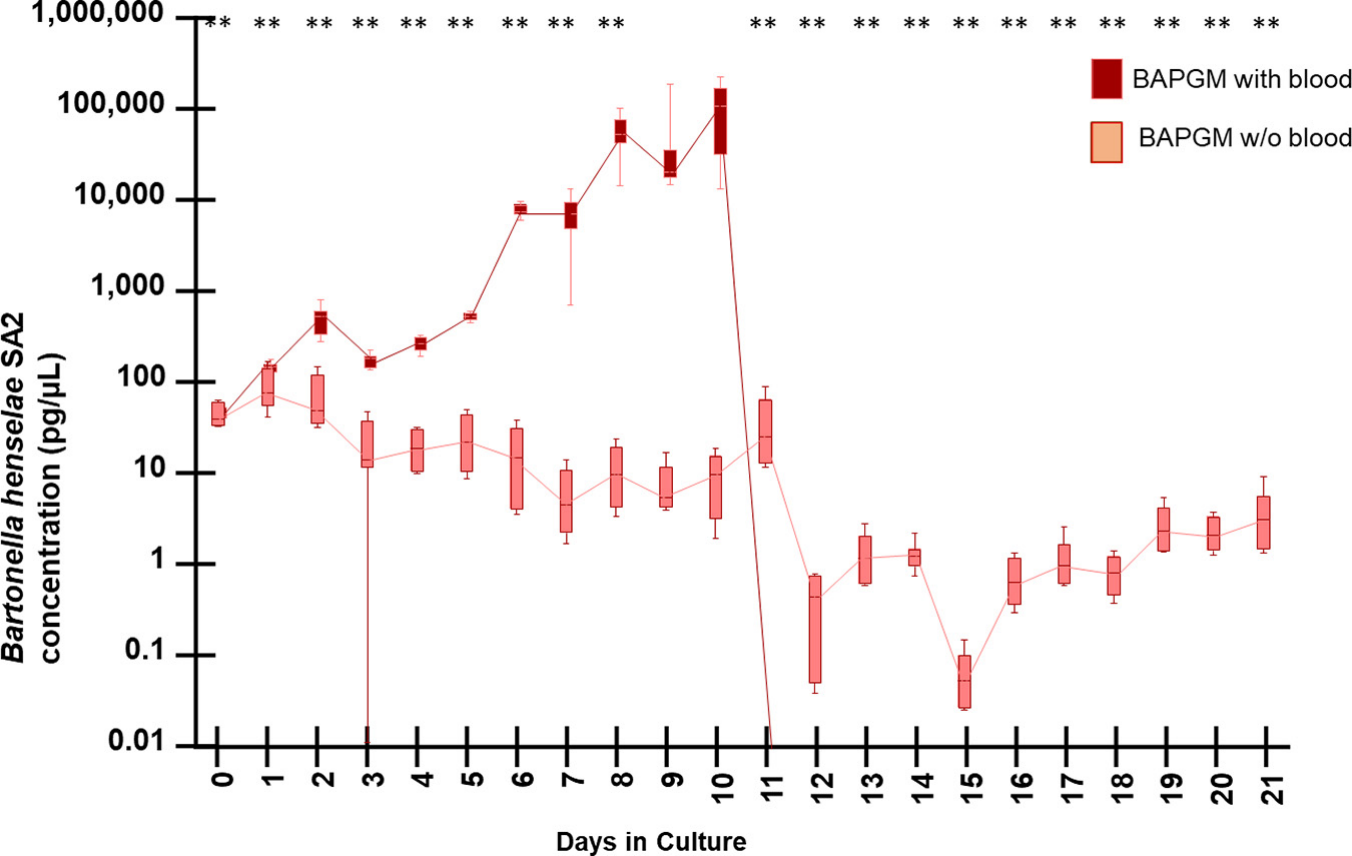
Growth kinetics determined by qPCR amplification of B. henselae SA2 DNA when cultured in BAPGM medium supplemented with or without sheep blood supplementation. DNA extractions used for qPCR testing in this figure were generated using the QIAsymphony DSP DNA kit. Box and whiskers plot displaying variation in the median, the first and third quartiles, and the minimum and maximum values obtained. ** denotes *P* < 0.01 values.

To determine if the addition of sheep blood affects *Bh* SA2 growth in either medium, bacterial DNA quantification graphs using Brugge supplemented with and without sheep blood (Fig. 1) and BAPGM media supplemented with and without sheep blood (Fig. 2) were compared.

Based upon visual inspection and *P* values (Fig. 1), there was a significant reduction of *Bartonella* DNA amplification when B. henselae SA2 was cultured in Brugge with sheep blood supplementation compared to Brugge without blood, through the first 8 days of growth. After day 9, B. henselae SA2 DNA amplification was significantly higher (an order of magnitude on average) when the bacteria were grown in Brugge media with blood supplementation compared to without blood.

In contrast, substantially different growth kinetics were observed when B. henselae SA2 was grown in BAPGM with or without blood supplementation. Based upon visual inspection, (Fig. 2), *Bh* SA2 DNA concentration decreased over time (from the initial value at time zero) when the bacteria were inoculated into BAPGM without blood supplementation. When *Bh* SA2 was inoculated into BAPGM supplemented with blood, there was an increase in amplified bacterial DNA in cultures over time from day 0 to day 10. Subsequently, from day 11 to day 21, there was no B. henselae DNA amplification from BAPGM supplemented with blood, when using the QIAsymphony DSP DNA extraction kit.

**FIG 3:**
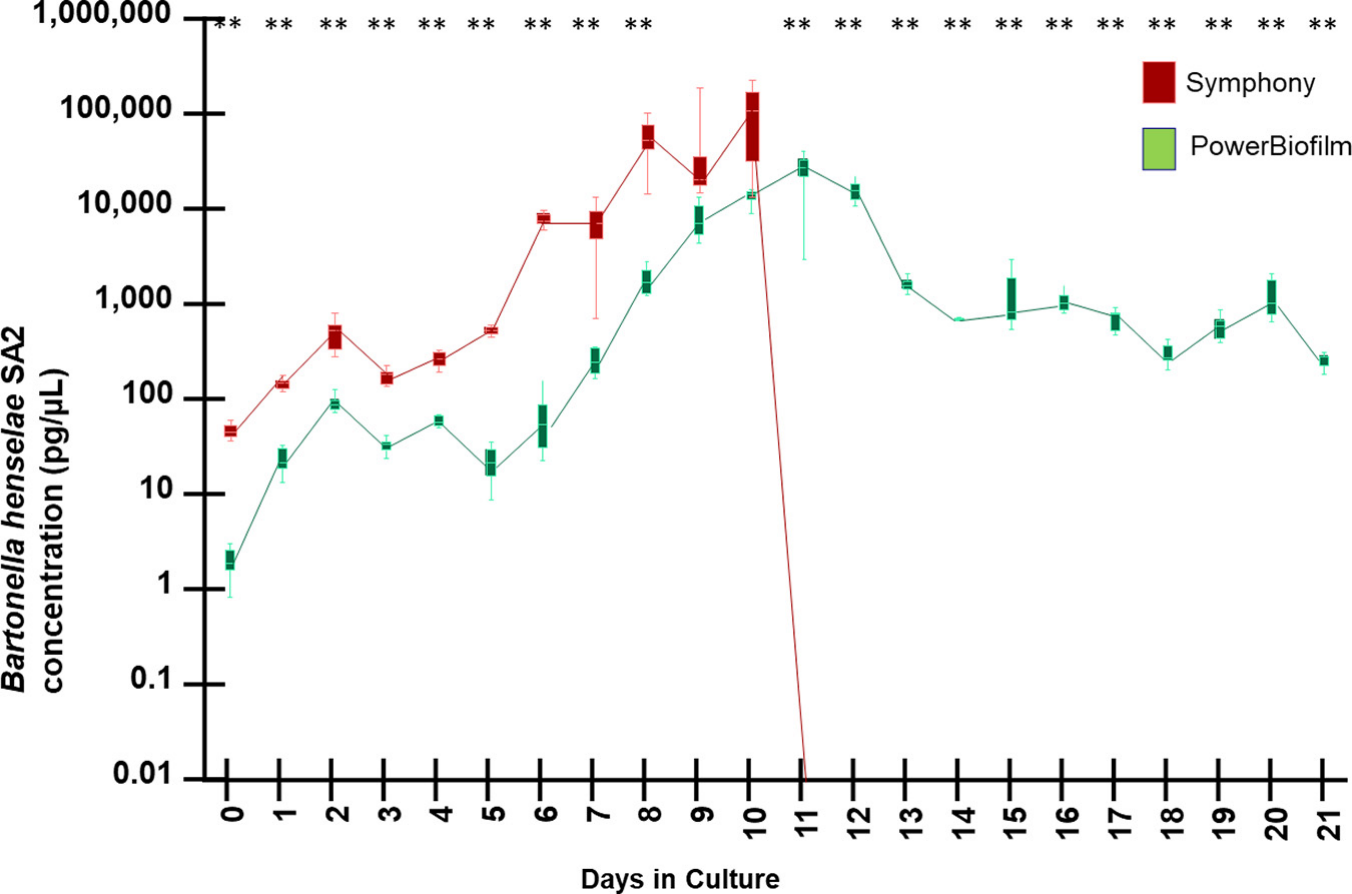
Growth kinetics determined by qPCR amplification of B. henselae SA2 DNA cultured in BAPGM with sheep blood supplementation using QIAsymphony and Qiagen DNeasy PowerBiofilm kits. Box and whiskers plot displaying variation in the median, the first and third quartiles, and the minimum and maximum values obtained. ** denotes *P* < 0.01 values.

In a prior experiment (data not shown), we found a nearly identical dramatic decrease in *Bh* SA2 DNA beginning at approximately the same postinoculation time point (day 11) and persisting through day 21. However, inoculation from day 14 and day 17 qPCR negative cultures onto blood agar plates (5% sheep blood TSAII agar plates, Sigma-Aldrich Burlington) resulted in visible *Bh* SA2 growth, with colony identity confirmed by qPCR amplification and DNA sequencing. To determine if the lack of *Bh* SA2 DNA amplification between postinoculation days 11 and 21 was related to biofilm formation, aliquots of the same blood-supplemented BAPGM culture samples from day 0 to 21 were extracted using the PowerBiofilm method (Fig. 3).

As seen in Fig. 3, when BAPGM with blood supplementation samples were extracted using the DNeasy PowerBiofilm Kit, amplification of *Bh* SA2 DNA was detectable from day 0 to day 21. The consistent qPCR amplification of *Bh* SA2 DNA during the entire 21-day culture period, as observed by samples extracted using the PowerBiofilm method, was suggestive of biofilm formation resulting in inefficient DNA extraction using the automated QIAsymphony DNA extraction method or alternatively the presence of an inhibitor that interfered with qPCR amplification.

### Documentation of PCR inhibition.

**FIG 4:**
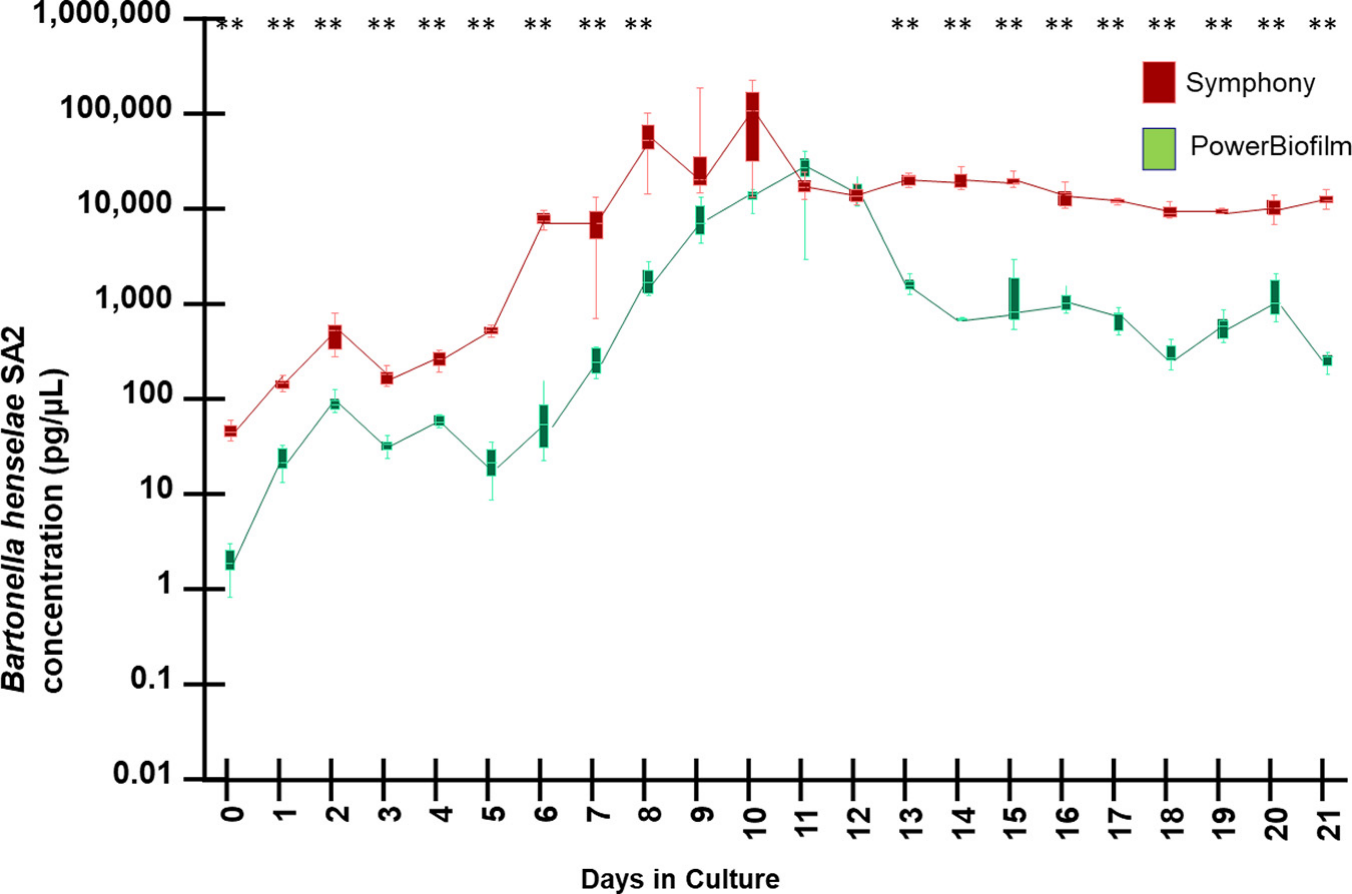
Growth kinetics determined by qPCR amplification of B. henselae SA2 DNA cultured in BAPGM with sheep blood supplementation using QIAsymphony and a 1:100 dilution with molecular grade water to counteract DNA inhibition, compared to the Qiagen DNeasy PowerBiofilm DNA extraction. Box and whiskers plot displaying variation in the median, the first and third quartiles, and the minimum and maximum values obtained. ** denotes *P* < 0.01 values.

There was no droplet digital PCR amplification of *Babesia* DNA in any of the spiked DNA samples (results not shown), supporting the presence of an inhibitor of DNA amplification. When the original QIAsymphony-extracted DNA samples from BAPGM with blood culture, days 11 to 21, were diluted in molecular grade water at a 1:100 ratio, *Bh* SA2 DNA was successfully amplified from all the previously negative samples (Fig. 4).

When qPCR inhibition was corrected by dilution of the extracted DNA with molecular grade water, the mean concentration of *Bh* SA2 growth in BAPGM supplemented with sheep blood progressively increased, with concentrations reaching 3 orders of magnitude greater than the time zero value by days 8 to 10 of culture and remaining high through the end of the experiment on day 21.

At all culture time points, and regardless of which of the 2 DNA extraction methods were used, the growth of *Bh* SA2 in BAPGM supplemented with sheep blood (Fig. 4) was significantly higher than when the bacteria were grown in BAPGM without blood supplementation (Fig. 2). *P* values for specific time points included: *P* = 0.0099 at day 7, *P* < 0.0001 at day 14, and *P* < 0.0001 at day 21.

### Blood supplementation in BAPGM and Brugge.

**FIG 5:**
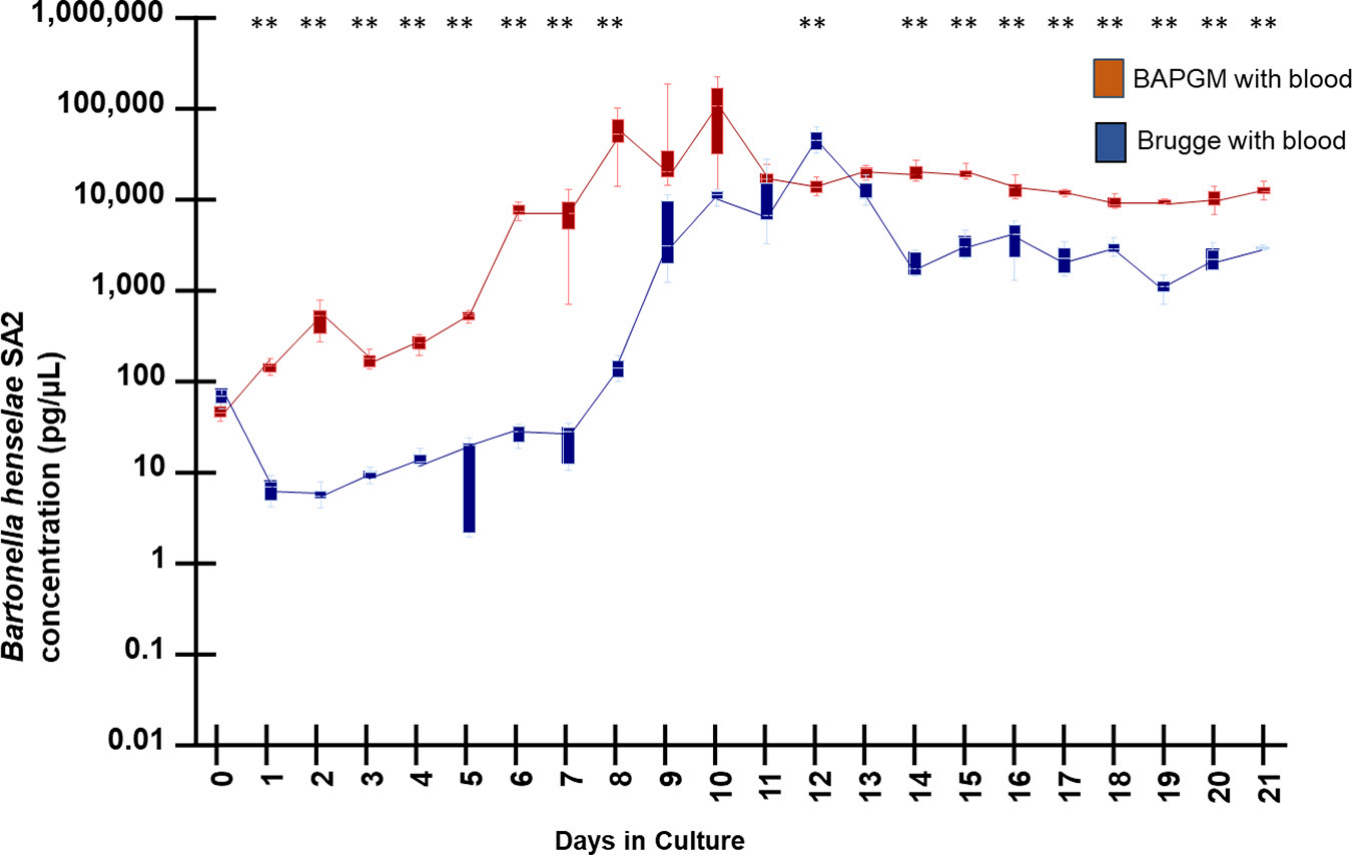
DNA kinetics determined by qPCR amplification of B. henselae SA2, when cultured in BAPGM (extracted DNA diluted 1:100 in molecular grade water) and Brugge medium supplemented with sheep blood. Box and whiskers plot displaying variation in the median, the first and third quartiles, and the minimum and maximum values obtained. ** denotes *P* < 0.01 values.

Comparing both media supplemented with sheep blood (Fig. 5), *Bh* SA2 concentrations were significantly higher at 17 of the 21 time points. On culture day 7, there was a substantial difference (357 times) in growth B. henselae DNA concentration between the 2 media (6,791 pg/μL for BAPGM versus 19 pg/μL for Brugge, *P* = 0.01). On day 8, there was a 418 times difference in growth between the 2 media (54,804 pg/μL for BAPGM versus 131 pg/μL for Brugge, *P* = 0.001). In contrast, there was only a single order of magnitude difference between the 2 media between culture days 14 and 21.

## DISCUSSION

Due to the increasing clinical importance in chronically ill immunocompetent, as well as immunocompromised individuals, Bartonella henselae has become a diagnostically relevant pathogen in recent years (19, 20, 24, 25, 27, 28, 34, 37, 44, 47, 70, 72, 97, 98). Due to a lack of sensitivity, culture isolation and PCR amplification of *Bartonella* spp. DNA from human patient specimens remain problems for clinical diagnostic laboratories. Because *Bartonella* spp. are present at levels in blood of non-reservoir hosts (dogs, horses, or humans) that often cannot be detected (either by molecular methods or by bacterial isolation), optimization of an enrichment medium (like BAPGM) in conjunction with more sensitive PCR amplification technologies is of substantial diagnostic importance (47, 50, 51, 63, 65, 66). Enrichment culture prior to DNA extraction, PCR amplification, or subculture isolation has proven to be essential for the detection of these pathogenic bacteria in asymptomatic people (47, 50, 51, 55, 70), in patients with neurological symptoms (17–19, 34, 97), and in healthy blood donors (63, 65, 66).

BAPGM, as an enrichment media for *Bartonella* culture, has been used by several research laboratories for the documentation of *Bartonella* species infections in animals and people (10, 33, 47, 50, 51, 63, 65, 66, 97, 99). To our knowledge, BAPGM is currently the only enrichment culture media specifically used for the diagnosis of bartonelloses in animals and humans in a CLIA accredited laboratory.

Previous studies have identified enhanced bacterial growth using insect cell culture media (52, 60–66). A primary goal of this study was to determine if supplementation of sheep blood, when added to the BAPGM *Bartonella* spp. enrichment blood culture platform or when added to a mammalian-based cell culture growth medium resulted in enhanced detection of bacterial DNA. We found that the growth kinetics differed between the 2 media: the addition of sheep blood improved *Bh* SA2 DNA detection throughout the 21 day culture period for BAPGM but resulted in a transient decrease in bacterial DNA concentration following inoculation of the Brugge media. Regardless of the basic media used (insect or mammalian cell culture-based), after approximately 12 days, the growth of *Bh* SA2 at 36°C, 5% CO_2_ and 100% humidity was strongly enhanced by the addition of sheep blood as a supplement.

The B. henselae DNA quantification trends for BAPGM and Brugge media, when supplemented with sheep blood, were comparable in supporting *Bh* SA2 growth. However, as evaluated in this study, BAPGM supplemented with sheep blood further enhanced the growth of *Bh* SA2 compared to supplemented or unsupplemented Brugge media. BAPGM generated significantly higher DNA amplification concentrations compared with Brugge media, as observed at culture days 7, 14, and 21 (the designated culture DNA extraction days that are routinely assessed in our laboratory for the detection of *Bartonella* spp DNA during human clinical research diagnostic testing) (17–24, 27–29, 31, 34, 37, 44, 45, 47, 48, 50, 51, 55, 70, 72, 97, 100–103). It is important to emphasize that the same *Bh* SA2 inoculum was used in a consistent manner to evaluate both media; however, the quantity of bacteria used in this study was substantially greater than the quantity of bacteria in healthy or sick immunocompetent humans. Therefore, when culturing patient blood specimens, the DNA quantification kinetics for both media might differ from the results of this study.

Initially, we considered 2 potential reasons for the lack of DNA amplification for samples extracted by the QIASymphony system following the peak growth of *Bh* SA2 in BAPGM. Among other potential explanations, either the presence of a DNA polymerase inhibitory compound (potentially co-eluted during DNA extraction) or a process related to rapid induction of biofilm formation when *Bh* SA2 reached maximal growth were considered most likely. PowerBiofilm DNA extraction in conjunction with *Bh* SA2 qPCR results clearly indicated that bacterial DNA persisted after maximal growth was achieved. Although concurrent biofilm formation cannot be ruled out, based upon dilution studies, a DNA amplification inhibitory compound/s seems to be generated when *Bh* SA2 growing in BAPGM medium supplemented with sheep blood reaches culture day 10 (coincident with the maximum growth time point). This DNA amplification inhibitory compound was not detectable when *Bh* SA2 growth was achieved at similar levels in Brugge media supplemented with sheep blood, or when the bacteria was grown in unsupplemented BAPGM or Brugge media. To further analyze this observation, our laboratory is currently attempting to identify and characterize the nature of this PCR inhibition.

The Bartonella henselae SA2 strain selected for these experiments was isolated from a human clinical case (44). We used a low passage isolate (passage 4), as this species has been shown to undergo phase variation after multiple laboratory passages in association with decreased auto agglutination, cell adherence, and potentially, decreased virulence (104–110). Whether the *Bh* SA2 results can be extrapolated to other *Bartonella* spp., other *Bh* strains at the same or lower levels of *in vitro* inoculation, or the growth kinetics generated from bacteremic patient blood specimens is an unknown limitation of this *in vitro* comparative culture study.

In this study, we were unable to produce growth kinetic curves of B. henselae via CFU enumeration. This was due to the difficulties associated to obtaining visible colonies (i.e., by subculturing from liquid media onto agar plates) and the length of time (usually weeks) for *Bartonella* to form visible colonies (44, 47, 61, 111, 112). Based upon our experience, only 10% of viable bacteria growing in liquid broth can develop into visible colonies (data not published).

Despite these limitations, the diagnostic utility of the BAPGM enrichment culture is based upon the molecular detection of *Bartonella* specific DNA sequences after the bacteria grow to levels that can be detected by PCR. The utility and application of this platform has been previously well documented (18, 20, 22, 24, 25, 31, 37, 45–47, 50, 51, 68, 70–75).

Even though B. henselae is capable of infecting numerous host cell types, bacterial division is only known to occur within erythrocytes (113–117), potentially due a high heme dependence for bacterial growth (118–123). In fact, the addition of hemoglobin has proven necessary for *Bartonella* spp. survival, growth, and isolation (120, 122–126). Although our diagnostic research testing utilizes 2 mL of patient blood inoculated into 8 mL of BAPGM liquid culture media, supplementation with additional, un-infected sheep blood seems to be required to achieve optimal bacterial growth (unpublished data from IPRL). Whether essential nutrients are depleted or substances that inhibit bacterial growth occur in the blood of patients chronically infected with B. henselae is unknown. Future, clinically relevant diagnostic studies comparing sheep blood supplementation to unsupplemented patient blood inoculums are needed. The findings reported in this study will hopefully encourage additional research efforts to enhance the sensitivity of *Bartonella* spp. testing, particularly in patients experiencing chronic illnesses. Not only would enhanced direct detection sensitivity of *Bartonella* spp. facilitate patient management decisions, it will facilitate the accurate characterization of the pathogenic potential of these bacteria in association with specific diseases.

## MATERIALS AND METHODS

### Pathogen.

A laboratory strain of Bartonella henselae SA2 (*Bh* SA2) isolated from a human clinical case (44) was used as a model organism for assessing liquid culture performance. Frozen stock of *Bh* SA2 (fourth passage) was revived by blood agar plating and incubation at 35°C, 100% humidity, and 5% CO_2_ until colonies were observed. A single colony was selected and inoculated into 10 mL of Brugge media and cultured under the same conditions for up to 14 days. This culture was used as the inoculum to assess the influence of blood supplementation on the microbiological utility of BAPGM and Brugge media for *Bartonella* enrichment blood culture.

### Media, culture conditions, and sample collection.

BAPGM and Brugge media were used to assess the growth kinetics of the *Bh* SA2 inoculum. BAPGM was prepared as previously described (47, 50, 51, 53, 55, 69). Brugge media is a DMEM/Nutrient Mixture F-12 (DMEM/F12) based media that supports the growth of different mammalian cell types in culture (127–135). This DMEM based medium was chosen for its ability to support the growth of *Bartonella* species, both as intracellular (i.e., within Vero E6 cells) or as planktonic (61, 136–138) bacteria.

**FIG 6:**
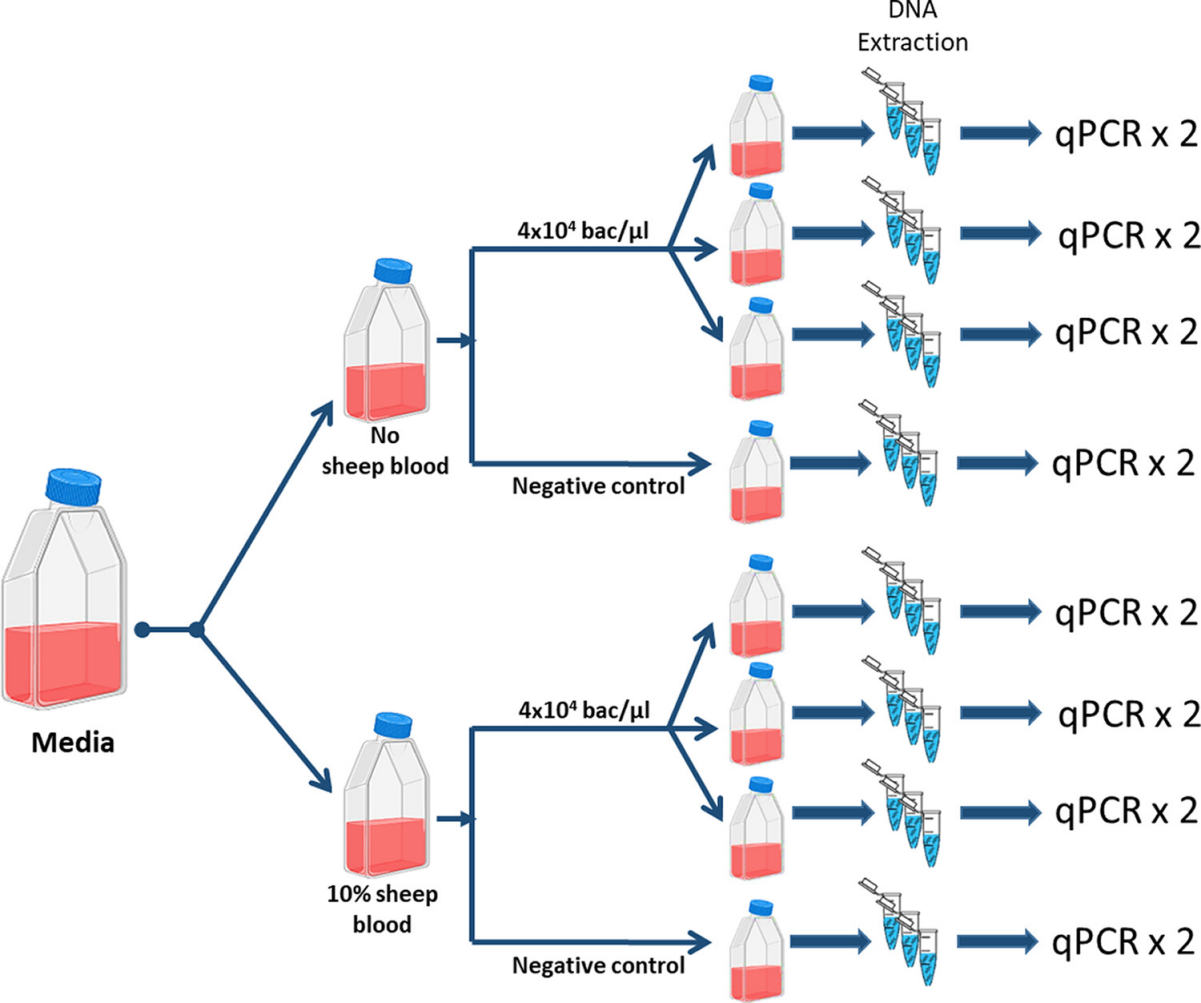
Experimental design using BAPGM and Brugge media, with or without sheep blood supplementation and inoculation with B. henselae SA2. All inoculated cultures were set up in triplicate. At each testing time point, 3 DNA extractions were performed per flask and each extraction tested in duplicate by qPCR. Naive cultures (a single flask per media/supplementation) were not inoculated with B. henselae SA2 and served as negative controls.

Bartonella henselae SA2 was inoculated into 10 mL of either BAPGM or Brugge media with or without 10% defibrinated sheep blood, at a final concentration of 4x10^4^ bacteria per microlliter (as determined by qPCR) (Fig. 6). Prior to use, sheep blood (Carolina Biological Supply Company, Sheep Blood Defibrinated, 100 mL) was exhaustively tested for potential infection with either B. henselae or *B. melophagi* according to previously published methods (139–143). All cultures, including BAPGM with blood, BAPGM without blood, Brugge without blood, and Brugge with blood, were performed in triplicate. A naive (uninoculated) culture control for each media with and without blood served as negative controls. Incubation was performed at 35°C, 100% humidity, and 5% CO_2_ with constant shaking.

Six aliquot samples of 200 μL each (3 for each DNA extraction method, see below) were collected from each culture flask every 24 h for 22 days. Aliquots were stored at −80°C until processed for DNA extraction.

### DNA extraction methods.

Two methods were used for DNA extractions: a QIAsymphony DSP DNA kit and the DNeasy PowerBiofilm Kit (both from Qiagen). The QIAsymphony DSP DNA kit, used for all bacterial and media DNA extractions, combines the speed and efficiency of silica-based purification of DNA with the convenient handling of magnetic particles in an automated liquid handler system (QIASymphony). The DNeasy PowerBiofilm kit, used only for the BAPGM enrichment culture with blood kinetic testing DNA extractions, is a column-based manual extraction method that uses a chemical lysis technique (Inhibitor Removal Technology [IRT]) that removes concentrated inhibitors typically present in biofilms.

### Quantitative PCR assay.

Quantitative PCR amplification of the *Bartonella* ITS region was performed as described elsewhere (10, 47, 49–51, 53) with minor modifications. Briefly, oligonucleotide primers BsppITS325s: 5′ CTT CAG ATG ATG ATC CCA AGC CTT CTG GCG 3′ and 543as: 5′ AAT TGG TGG GCC TGG GAG GAC TTG 3′ were used as forward and reverse primers, respectively. Each 25 μL PCR was prepared using 12.5 μL of SoAdvanced Universal Sybergreen Supermix (Bio-Rad), 0.2 μL of 100 μM each forward and reverse primers (IDT DNA Technology), 7.5 μL of Ultra-Pure, molecular grade water (Genesee Scientific), and 5 μL of DNA from each sample tested culture. Quantitative PCR was performed in an CFXOpus (Bio-Rad) under the following conditions: a single hot-start cycle at 95°C for 3 min followed by 45 cycles of denaturing at 94°C for 10 s, annealing at 68°C for 10 s, and extension at 72°C for 10 s. Amplification was completed by an additional cycle at 72°C for 30 s. Positive amplicons were analyzed by analysis of detectable fluorescence versus cycle threshold values, as well as by melting curve.

Each DNA sample was tested by qPCR in duplicate. The average cycle threshold value was converted into a DNA concentration by semi-logarithmic linear regression using standard *Bartonella* DNA concentrations ran at the same time and under the same thermocycler conditions.

**Evaluation for potential PCR inhibition.** Two approaches were used to assess the potential of qPCR inhibition in DNA samples extracted from BAPGM with blood during postinoculation days 11 through 21. First, the extracted DNA from these dates was spiked with Babesia microti DNA as an internal control, followed by *Babesia* DNA amplification using droplet digital PCR, as previously reported (58). For the second approach, we tested *Bartonella* DNA amplification by qPCR after each DNA sample was diluted using molecular grade water at a 1:100 ratio.

### Statistical analyses.

Raw Ct values were converted to a B. henselae log concentration by creating a Standard Curve based on positive control Ct values from the same agar plate as the inoculum. A y = mx+b approach was used, where x represented the Raw Ct values. To determine the B. henselae concentration, the log concentrations were then converted by taking the 10^(x) of the B. henselae log concentration.

Outliers were determined using the ROUT method at Q = 10% to remove the most likely outliers for each individual sample obtained from each flask tested. Once outliers were removed from the data set, mean B. henselae concentration for each of the 4 experimental conditions (BAPGM with blood, BAPGM without blood, Brugge with blood, and Brugge without blood) at each time point (*n* = 22) was calculated using the B. henselae log concentration from each of 18 replicates (3 flasks for each condition, 3 aliquots removed per day, and 2 qPCR replicates performed on each aliquot). *Bartonella* DNA quantification graphs were plotted for each experimental condition. The mean B. henselae concentrations for each media at each time point were then compared using a 2-way mixed effects ANOVA with a restricted maximum likelihood (REML) estimate. Values removed as outliers were considered as missing values. Sphericity was not assumed. Fixed effects of each comparison were the day of collection, media triplicates, and day of collection x media triplicates. Random effects included media groups and residuals. Sidak’s multiple comparison test was used to control for multiple comparisons; *P* values were considered significant at *P* < 0.05 unless otherwise specified. Graphical comparisons utilized box and whiskers plots displaying variation in the median, the first and third quartiles, and the minimum and maximum values obtained. Significance is denoted by ** for *P* < 0.01 values. All data analysis was performed using GraphPad Prism 9.4.1.
